# Central pancreatectomy of the remnant pancreas without reconstruction after pancreatoduodenectomy

**DOI:** 10.1186/s40792-024-02018-1

**Published:** 2024-09-11

**Authors:** Kinji Hirono, Kosei Takagi, Motohiko Yamada, Jiro Kimura, Tomokazu Fuji, Kazuya Yasui, Takeyoshi Nishiyama, Yasuo Nagai, Noriyuki Kanehira, Toshiyoshi Fujiwara

**Affiliations:** 1 Department of Gastroenterological Surgery, Okayama University Graduate School of Medicine, Dentistry, and Pharmaceutical Sciences, 2-5-1 Shikata-Cho, Kita-Ku, Okayama, 700-8558 Japan; 2 Department of Gastroenterological Surgery, Shobara Japan Red Cross Hospital, Hiroshima, Japan

**Keywords:** Central pancreatectomy, Pancreatoduodenectomy, No reconstruction, Glucose tolerance

## Abstract

**Background:**

There are several reports on the safety and feasibility of pancreatoduodenectomy (PD) without reconstruction of the small remnant pancreas. However, a few studies have explored central pancreatectomy (CP) for non-reconstructed small remnant pancreases after PD. This study presents a case of CP without pancreatic reconstruction after PD.

**Case presentation:**

A 58-year-old man with cerebral palsy underwent PD for distal cholangiocarcinoma. Three years postoperatively, a 12-mm tumor was detected in the remnant pancreatic body and diagnosed as a pancreatic neuroendocrine neoplasm. Surgical resection was performed, because the tumor was enlarged and chemotherapy resistant. The afferent loop with pancreatojejunostomy anastomosis was dissected, and CP, including pancreatojejunostomy anastomosis, was performed. Given the remnant pancreas was hard and atrophic, the pancreatic tail was transected using a stapler without reconstructing the small remnant pancreas. The patient experienced no postoperative complications including postoperative pancreatic fistula, and the endocrine function of the pancreas was preserved.

**Conclusions:**

We present a case of remnant pancreatic CP that did not require reconstruction after PD. Preservation of the small remnant pancreas without reconstruction during CP may be feasible to maintain endocrine function in select patients after PD.

## Background

Pancreatic reconstruction using a small pancreatic remnant after pancreaticoduodenectomy (PD) or central pancreatectomy (CP) is technically challenging. Although total pancreatectomy is an option in such cases, preserving the small remnant pancreas after PD can help maintain endocrine function [1]. To date, several studies have reported the safety and feasibility of PD without pancreatic reconstruction for small remnant pancreas [1–3]; however, few have examined CP without pancreatic reconstruction following PD. Herein, we present a case of CP without pancreatic reconstruction after PD.

## Case presentation

**Fig. 1:**
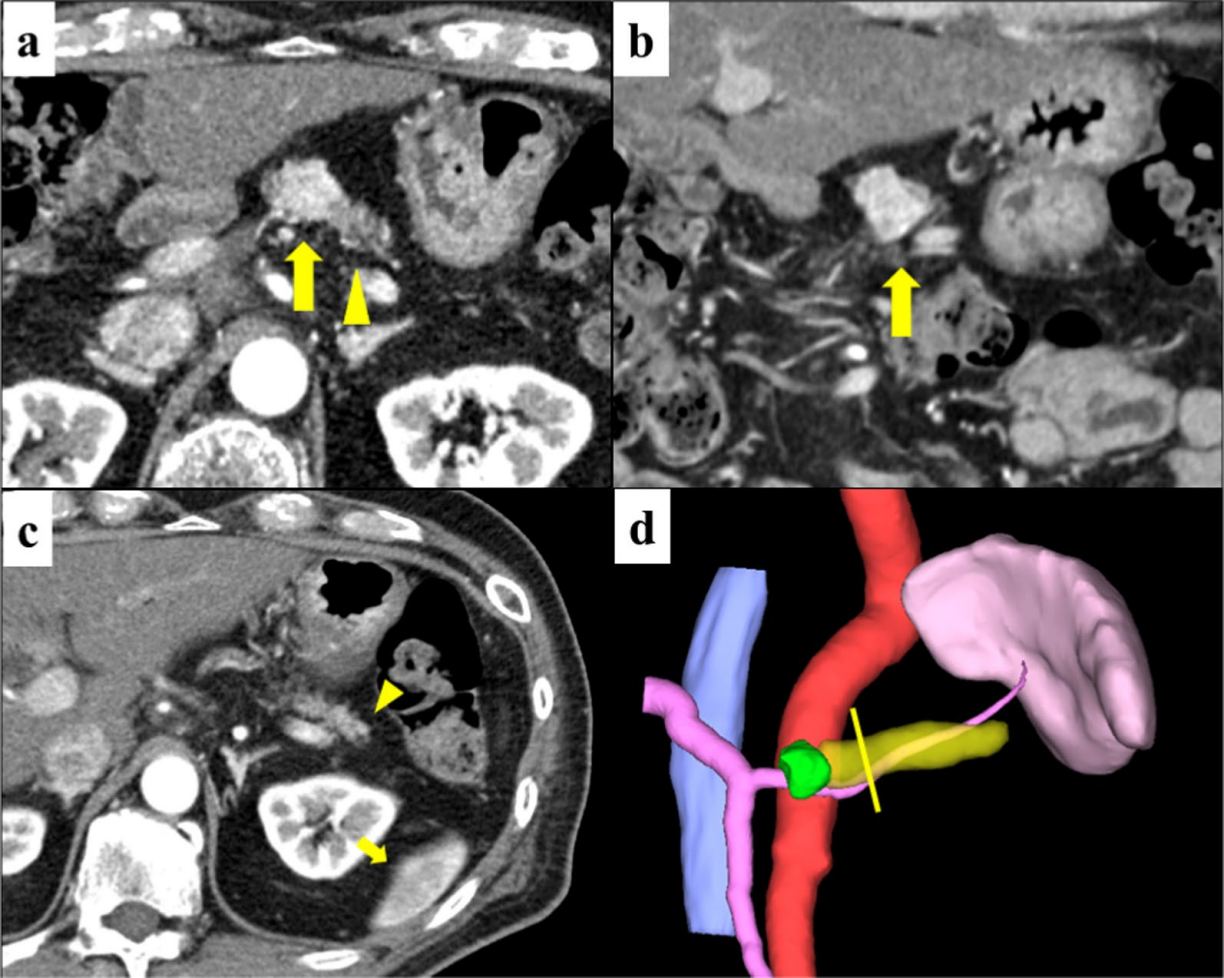
Contrast-enhanced abdominal computed tomography scan. **a** Axial view: The 19-mm tumor with contrast enhancement (arrow) is located at the pancreatic body near the pancreatojejunostomy site. The remnant pancreas is atrophic (arrowhead). **b** Coronal view. **c** Association between the distal edge of the pancreas (arrow) and the spleen (arrowhead).** d** 3D construction image (Synapse Vincent; green, tumor; yellow, remnant pancreas; yellow line, estimated transection line)

A 58-year-old man with a medical history of cerebral palsy and diabetes mellitus underwent subtotal stomach-preserving PD with modified Child’s reconstruction for distal cholangiocarcinoma (T4N0M0) [4]. During follow-up examinations every 3 months, the patient did not experience any recurrence. However, 3 years after PD, a 12-mm contrast-enhanced tumor was detected in the remnant pancreatic body on abdominal computed tomography (CT) images. Endoscopic ultrasound-guided fine-needle aspiration revealed a grade I pancreatic neuroendocrine neoplasm (PNEN) with grade 1 (G1). No metastatic lesions were detected by somatostatin receptor scintigraphy. Considering the patient’s social background, including cerebral palsy with mental retardation and living alone, surgical resection was not initially indicated. Chemotherapy with lanreotide acetate was initiated, and the tumor did not grow over the next five years. However, a recent CT scan revealed an increase in the tumor size from 12 to 19 mm (Fig. 1). The remnant pancreas was atrophic, with a volume of 7.6 mL calculated using a CT image analysis system (Synapse Vincent; Fujifilm Medical, Tokyo). Consequently, we decided to perform surgical resection of the CP through the multidisciplinary meeting. Informed consent was obtained from the patient preoperatively.

**Fig. 2:**
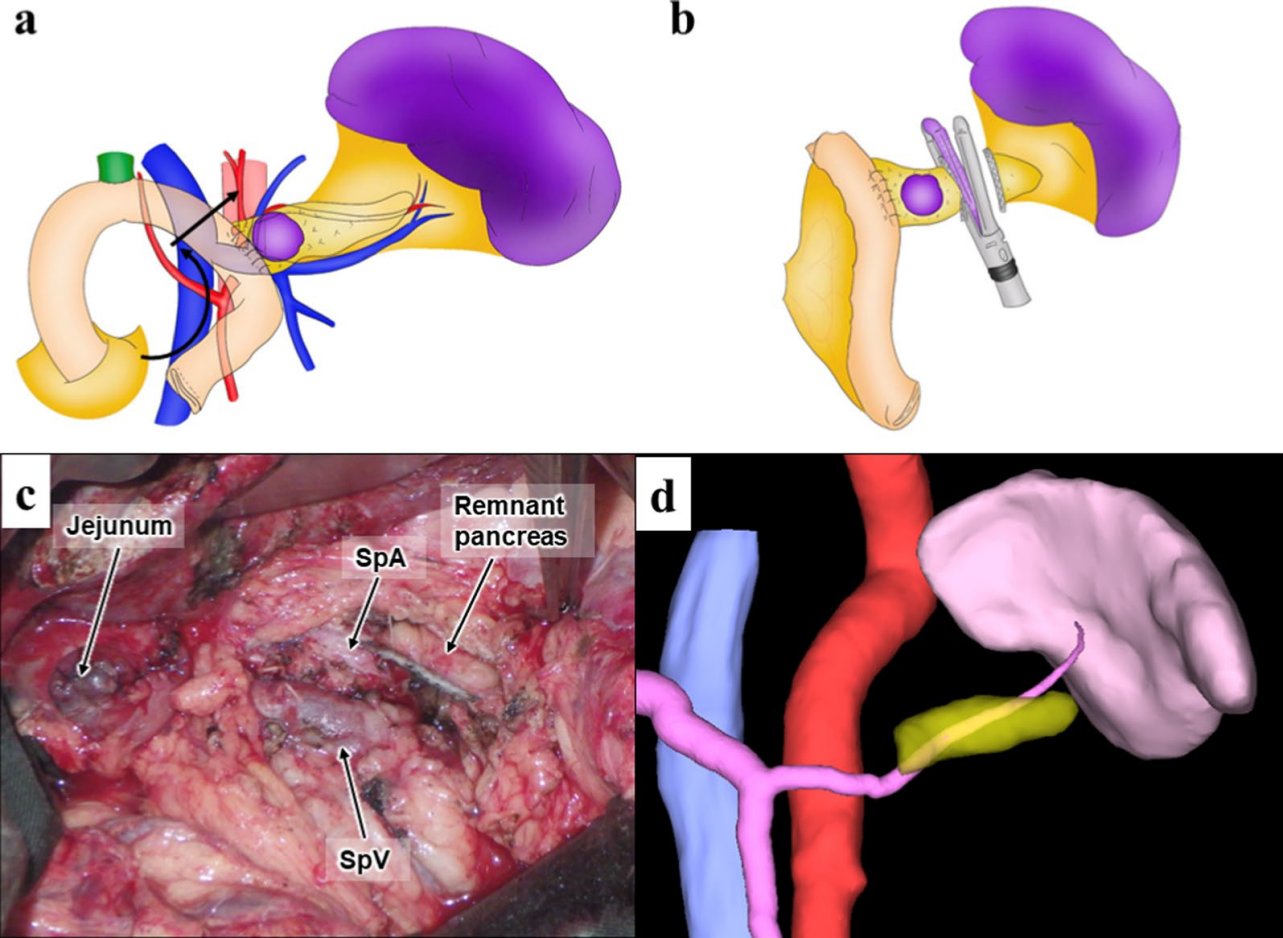
Operative findings. **a** Division of the jejunum and its mesentery (arrow). **b** The pancreatic tail transected using a reinforced stapler. **c** No reconstruction of the remnant pancreas was performed. **d** 3D construction image (Synapse Vincent) on 1 month after surgery. SpA, splenic artery; SpV, splenic vein

During surgery, a 2-cm tumor was palpable in the pancreatic body near the pancreatojejunostomy anastomosis site. The remnant pancreas is hard and atrophic. The afferent loop with pancreatojejunostomy anastomosis was dissected, and the jejunum was divided between the pancreatojejunostomy and hepaticojejunostomy anastomoses. The pancreatic body and surrounding tissues were dissected toward the pancreatic tail while preserving the splenic vessels. Subsequently, the pancreatic tail was transected using a reinforced stapler without reconstructing the small remnant pancreas (Fig. 2). The operative time and blood loss were 227 min and 310 mL, respectively.

**Fig. 3:**
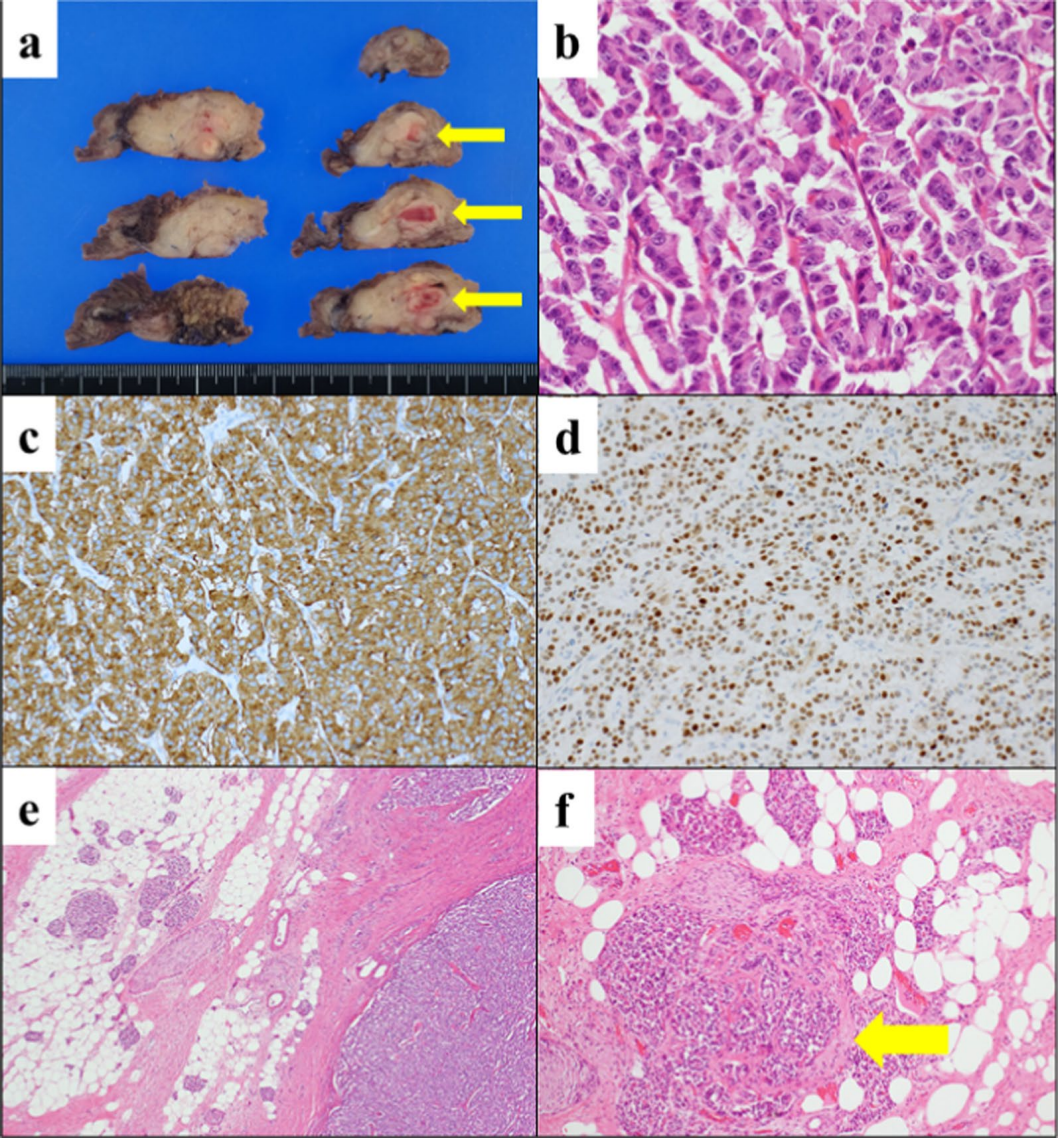
Specimens. **a** Macroscopic findings of the cut surface: The tumor located at the body of the pancreas near pancreatojejunostomy site (arrow). **b** Microscopic findings: Hematoxylin & Eosin (H&E) staining, 400 × magnification. Small round cells are proliferating in the tumor. **c** Synaptophysin positive staining. **d** Insulinoma-associated protein 1 positive staining. **e** H&E staining, 10 × magnification: Most of acinar cells in the pancreatic parenchyma replaced by adipose tissue. **f** H&E staining, 40 × magnification: Well-preserved islet cells (arrow)

Pathological findings revealed small round cells that had proliferated and infiltrated the vasculature. These cells were positive for synaptophysin (and) insulinoma-associated protein 1 ( +), a Ki-67 index of 2.8%, and mitosis in less than 1/10 high-power fields, consistent with PNEN G1. Additionally, the surgical margins were negative, the acinar cells disappeared, and the islet cells were well preserved (Fig. 3).

The postoperative amylase and drain amylase levels were 43 U/L and 34 U/L on postoperative day (POD) 1, 103 U/L and 71 on POD3, and 73 U/L and 61 U/L on POD 5. The drain was removed on POD 6. The patient was discharged on POD 10 without complications such as postoperative pancreatic fistula (POPF). The estimated remnant pancreatic volume was 6.2 mL 1 month after surgery (Fig. 2d). Fourteen months after surgery, the patient remains alive without recurrence. His hemoglobin A1c level remained unchanged at 6.2%, which was the same as the preoperative value. Body weight also remained stable, with a postoperative weight of 72.4 kg and a weight of 71.6 kg 14 months after surgery.

## Discussion

To the best of our knowledge, this is the first report of CP without remnant pancreatic reconstruction after PD in a patient with a PNEN G1. Preservation of a small remnant pancreas without reconstruction during CP can be safely performed in select cases after PD. Although total pancreatectomy after PD is technically simpler than CP, owing to the lack of the risk of POPF, CP after PD can secure endocrine function.

In this case, several factors influenced the decision to opt for CP instead of total pancreatectomy. Surgical resection, including enucleation, is recommended for nonfunctioning pancreatic neuroendocrine neoplasms < 2 cm in size [5]. In this case, the tumor was adjacent to the pancreatojejunostomy anastomosis site, making enucleation impractical; however, CP was considered oncologically safe. Given the social background, he was unable to receive insulin injections because of cerebral palsy and mental retardation.

Several factors should be considered when using a non-reconstruction technique for the remnant pancreas. Although repeated pancreatojejunostomy is the first option, another Roux-en-Y loop must be created by dissecting intra-abdominal adhesions in patients after PD. Moreover, the main pancreatic duct may not be visible in the atrophic remnants of the pancreas. In such cases, repeated pancreatic enterostomies are technically challenging. By contract, a major concern regarding CP using non-reconstruction techniques is the risk of POPF. Once the pancreatic stump opens, there is a potential risk of refractory POPF. However, pancreatic exocrine insufficiency commonly after PD [6]. Furthermore, a recent study reported a low incidence of POPF in patients with non-reconstructed small remnant pancreas after PD [1]. Therefore, we suggest that the risk of POPF after CP with a non-reconstructed small remnant pancreas following PD was low in the present case. Despite the presence of small remnants, long-term pancreatic endocrine function is well preserved [1]. For patients undergoing total pancreatectomy, lifelong insulin injection therapy is required; therefore, preserving a small remnant pancreas should be considered. A previous report revealed that islet cells in the hard pancreatic body/tail are well preserved even after severe fibrosis and the disappearance of acinar cells in patients who underwent PD [7]. Microscopic findings of the pancreatic parenchyma in the present case support these findings (Fig. 3e, f. Exocrine acinar cells are diminished and replaced with adipose tissue. However, the endocrine islet cells were preserved, supporting the preservation of endocrine function in the hard pancreas. Although endocrine function was preserved postoperatively, further follow-up is required to assess the alterations in postoperative endocrine function.

## Conclusions

We present a case of CP in the remnant pancreas without reconstruction after PD. Preservation of the small remnant pancreas without reconstruction during CP may be feasible for securing endocrine function in selected cases after PD.

## Data Availability

None.

## Abbreviations

PD: Pancreatoduodenectomy
CP: Central pancreatectomy
PNEN: Pancreatic neuroendocrine neoplasm
CT: Computed tomography
POPF: Postoperative pancreatic fistula

## References

[1] Miyashita M et al., Low incidence of pancreatic fistula and well-preserved endocrine function with non-reconstructed small remnant pancreas after pancreaticoduodenectomy*.* Ann Gastroenterol Surg. 2024. 10.1002/ags3.12795 PMC11368497 39229551

[2] Toyama N, et al. Pancreas head carcinoma with total fat replacement of the dorsal exocrine pancreas. J Gastroenterol. 2004;39(1):76–80. 14767740 10.1007/s00535-003-1250-4

[3] Fujino Y, et al. Pancreaticoduodenectomy without reconstruction of remnant pancreas for pancreas tumors with acquired fatty replacement of distal pancreas. Hepatogastroenterology. 2007;54(77):1589–90. 17708307

[4] Takagi K, et al. Radiographic sarcopenia predicts postoperative infectious complications in patients undergoing pancreaticoduodenectomy. BMC Surg. 2017;17(1):64. 28549466 10.1186/s12893-017-0261-7 PMC5446724

[5] Ito T, et al. JNETS clinical practice guidelines for gastroenteropancreatic neuroendocrine neoplasms: diagnosis, treatment, and follow-up: a synopsis. J Gastroenterol. 2021;56(11):1033–44. 34586495 10.1007/s00535-021-01827-7 PMC8531106

[6] Pathanki AM, et al. Pancreatic exocrine insufficiency after pancreaticoduodenectomy: current evidence and management. World J Gastrointest Pathophysiol. 2020;11(2):20–31. 32318312 10.4291/wjgp.v11.i2.20 PMC7156847

[7] Yamaguchi T, et al. Histometric analysis of the distal pancreas in pancreatic head cancer. Surg Today. 1997;27(5):420–8. 9130344 10.1007/BF02385705

